# Emerging mechanisms of fluoroquinolone resistance.

**DOI:** 10.3201/eid0702.010239

**Published:** 2001

**Authors:** D C Hooper

**Affiliations:** Massachusetts General Hospital, Harvard Medical School, Boston, Massachusetts 02114-2696, USA. dhooper@partners.org

## Abstract

Broad use of fluoroquinolones has been followed by emergence of resistance, which has been due mainly to chromosomal mutations in genes encoding the subunits of the drugs' target enzymes, DNA gyrase and topoisomerase IV, and in genes that affect the expression of diffusion channels in the outer membrane and multidrug-resistance efflux systems. Resistance emerged first in species in which single mutations were sufficient to cause clinically important levels of resistance (e.g., Staphylococcus aureus and Pseudomonas aeruginosa). Subsequently, however, resistance has emerged in bacteria such as Campylobacter jejuni, Escherichia coli, and Neisseria gonorrhoeae, in which multiple mutations are required to generate clinically important resistance. In these circumstances, the additional epidemiologic factors of drug use in animals and human-to-human spread appear to have contributed. Resistance in Streptococcus pneumoniae, which is currently low, will require close monitoring as fluoroquinolones are used more extensively for treating respiratory tract infections.


### Emerging Mechanisms of Fluoroquinolone Resistance

**David C. Hooper**
Massachusetts General Hospital, Harvard Medical School, Boston, Massachusetts, USA

Broad use of fluoroquinolones has been followed by emergence of resistance, which has been due mainly to chromosomal mutations in genes encoding the subunits of the drugs' target enzymes, DNA gyrase and topoisomerase IV, and in genes that affect the expression of diffusion channels in the outer membrane and multidrug-resistance efflux systems. Resistance emerged first in species in which single mutations were sufficient to cause clinically important levels of resistance (e.g., *Staphylococcus aureus* and *Pseudomonas aeruginosa*). Subsequently, however, resistance has emerged in bacteria such as *Campylobacter jejuni*, *Escherichia coli*, and *Neisseria gonorrhoeae*, in which multiple mutations are required to generate clinically important resistance. In these circumstances, the additional epidemiologic factors of drug use in animals and human-to-human spread appear to have contributed. Resistance in *Streptococcus pneumoniae*, which is currently low, will require close monitoring as fluoroquinolones are used more extensively for treating respiratory tract infections.

The fluoroquinolone class of antimicrobial agents has had broad acceptance in hospitalized and community patients, and usage appears to be increasing (1,2). Although some members of the class (temafloxacin, grepafloxacin, and trovafloxacin) have been withdrawn or restricted because of adverse events, new members continue to be developed and approved (gatifloxacin and moxifloxacin). The last six released fluoroquinolones are for treating patients with respiratory tract infections, the single most common group of infections (3). This fact, plus the convenience of fluoroquinolones (once or twice a day oral dosing), suggests that use will increase (1).

As we approach the halfway point of the second decade of fluoroquinolone use, resistance has already emerged in some species of bacteria and some clinical settings. We examine the mechanisms of fluoroquinolone resistance and discuss epidemiologic factors that may have contributed to the prevalence of antibiotic resistance in clinical settings.

### Mechanism of Fluoroquinolone Action

Fluoroquinolones (and earlier quinolones) are novel among antimicrobial agents in clinical use because they directly inhibit DNA synthesis. Inhibition appears to occur by interaction of the drug with complexes composed of DNA and either of the two target enzymes, DNA gyrase and topoisomerase IV. These enzymes are structurally related to each other, both being tetrameric with pairs of two different subunits. The GyrA and GyrB subunits of DNA gyrase are respectively homologous with the ParC and ParE subunits of topoisomerase IV. Both enzymes are type 2 topoisomerases, which act by breaking both strands of a segment of DNA, passing another segment through the break, and then resealing the break. For DNA gyrase, this topoisomerization reaction results in introduction (or removal) of DNA supercoils, thus affecting the negative supercoiling of DNA necessary to initiate DNA replication and remove positive supercoils that accumulate before an advancing replication fork. For topoisomerase IV, the topoisomerization reaction results in separation of the interlocking of daughter DNA strands that develop during replication; this facilitates the segregation of daughter DNA molecules into daughter cells. In both cases, fluoroquinolones appear to trap the enzyme on DNA during the topoisomerization reaction, forming a physical barrier to the movement of the replication fork (4), RNA polymerase (5), and DNA helicase (6). The collision of the replication fork with these trapped complexes triggers other poorly defined events within the cell that ultimately result in cell death.

### Mechanisms of Fluoroquinolone Resistance

In all species studied, mechanisms of fluoroquinolone resistance include one or two of the three main mechanistic categories, alterations in the drug target, and alterations in the permeation of the drug to reach its target. No specific quinolone-modifying or -degrading enzymes have been found as a mechanism of bacterial resistance to fluoroquinolones, although some fungi can degrade quinolones by metabolic pathways (7).

### Alterations in Target Enzymes

Most extensively studied have been alterations in target enzymes, which are generally localized to specific domains of each subunit type. These alterations arise from spontaneous mutations in the genes encoding the enzyme subunits and thus can exist in small numbers (1 in $10^6$ to 1 in $10^9$ cells) in large bacterial populations. With GyrA and ParC subunits of resistant bacteria, amino acid changes are generally localized to a region of the enzyme in the amino terminus that contains the active site, a tyrosine that is covalently linked to the broken DNA strand during enzyme action. Resistance-causing amino acid changes are also clustered in three

Address for correspondence: David C. Hooper, Division of Infectious Diseases, Massachusetts General Hospital, 55 Fruit Street, Boston, MA 02114-2696, USA; fax: 617-726-7416; e-mail: dhooper@partners.org dimensions, based on the structure of a fragment of GyrA that has been solved by x-ray crystallography, suggesting that this region constitutes part of a quinolone-binding site on the enzyme (8). One of the common resistance mutations in GyrA, which causes a change from serine at position 83 to tryptophan, causes reduced binding of norfloxacin to the gyrase-DNA complex (9).

For the GyrB and ParE subunits of resistant bacteria, amino acid changes, when present (mutations in these subunits are much less common than those in GyrA or ParC), are usually localized to the mid-portion of the subunit in a domain involved in interactions with their complementary subunits (GyrA and ParC, respectively). The original crystal structure of yeast topoisomerase II, which is related to DNA gyrase and topoisomerase IV, had suggested that this resistance-determining domain was not in proximity to the resistance-determining domains of GyrA and ParC (10); however, structures of other enzyme conformations suggested that the resistance-determining regions of both types of subunits might be in proximity during certain parts of the enzyme catalytic cycle, perhaps defining an enzyme conformation to which quinolones bind (11). No crystal structures in which a quinolone is bound to the enzyme-DNA complex have been solved; thus, contact points between drug, enzyme, and DNA have not been directly determined. Also unknown is how different amino acid changes effect resistance.

### Differences in Fluoroquinolone Targets and Resistance

The interaction of a fluoroquinolone with the complexes of either DNA gyrase or topoisomerase IV with DNA may block DNA synthesis and result in cell death (12). The antibacterial potency of a quinolone is defined in part by its potency against the two enzyme targets; the more sensitive of the two enzymes within a cell is the primary target. Many fluoroquinolones have differing potencies against DNA gyrase and topoisomerase IV. A general pattern for most quinolones has emerged: DNA gyrase is the primary drug target in gram-negative bacteria, and topoisomerase IV is the primary target in gram-positive bacteria. These differences correlate with relative drug sensitivities in several cases, the more sensitive of the two enzymes being the primary target defined by genetic tests (13-15). The first step in mutational resistance in the drug target usually occurs by an amino acid change in the primary enzyme target, with a rise in MIC of the cell predicted to be determined by the effect of the mutation itself or by the level of intrinsic sensitivity of the secondary drug target (whichever is lower). Higher levels of resistance may then occur by second mutational steps, in which amino acid changes are selected in the secondary target enzyme. Further mutations result in additional amino acid changes in either enzyme, depending on which was least resistant in the cell under selection. On mechanistic grounds, this pattern of stepwise mutations in alternating target enzymes implies that both high intrinsic potency against the primary target and the similarity of potency against both targets will affect the likelihood of selection of first-step resistant mutants. Thus, fluoroquinolones with a high therapeutic index (defined as the concentration of drug at the site of infection divided by the MIC of the drug for the target bacterium), in which drug concentration exceeds the MIC of a first-step mutant, are unlikely to select spontaneous first-step mutants present in the infecting bacterial population; such mutants are inhibited or killed by these concentrations. Furthermore, the greater the extent to which a fluoroquinolone has similar (and ultimately equal) potency against both enzyme targets, the lower the MIC increment for a first-step drug target mutant. Thus, for drugs with low increments in resistance for first-step mutants because of similar activities against both target enzymes, the extent to which drug concentrations can exceed the MIC of first-step mutants may be enhanced. These principles would predict that selection of fluoroquinolone resistance could occur readily with ciprofloxacin against species such as *Staphylococcus aureus* and *Pseudomonas aeruginosa*, organisms in which single mutations cause MICs of ciprofloxacin that approach or exceed achievable serum concentrations. This prediction has been borne out by surveillance data (16).

### Alterations in Drug Permeation

To reach their targets in the cell cytoplasm, fluoroquinolones must cross the cytoplasmic membrane and, in gram-negative bacteria, the outer membrane as well. Fluoroquinolones are sufficiently small and have charge characteristics that allow them to cross the outer membrane through porin proteins, which form general diffusion channels; they also appear to cross the cytoplasmic membrane by diffusion (17). Resistance to fluoroquinolones in gram-negative bacteria is associated with reductions in porins and reduced bacterial accumulation of drug, but measurements of diffusion rates suggest that porin reductions alone are generally not sufficient to account for resistance (18).

More recently, resistance caused by reduced accumulation has been shown to require the presence and enhanced expression of endogenous efflux systems that actively pump drug from the cytoplasm. In gram-negative bacteria, these systems typically have three components: the efflux pump located in the cytoplasmic membrane, an outer membrane protein, and a membrane fusion protein thought to link the two. Drug is actively extruded from the cytoplasm or cytoplasmic membrane across the periplasm and outer membrane to the cell exterior; the energy for this process is derived from the proton gradient across the membranes. Pumps of this type also exist in gram-positive bacteria, and increased amounts of these pumps have been associated with low levels of fluoroquinolone resistance. These efflux systems are typically capable of causing resistance to compounds of diverse structural types and thus are referred to as multidrug resistance (MDR) pumps. They appear to be present in many if not all bacteria. The natural substrates for these systems are generally unknown, but current models envision a general role in removing toxic compounds from the cytoplasm or cytoplasmic membrane (19). Although fluoroquinolones are synthetic antimicrobial agents, a number of them are substrates for a range of efflux systems. Among pathogenic bacteria, *Escherichia coli*, *P. aeruginosa*, *S. aureus*, and *Streptococcus pneumoniae* have been most extensively studied for efflux systems causing fluoroquinolone resistance (Table). In most cases, expression of the components of the efflux system is regulated, and resistance occurs by chromosomal mutation that causes coordinated increased expression of pump components. The conditions under which there is physiologically increased expression of these systems are largely unknown. In *P. aeruginosa,* four such efflux systems have been identified, each differing by which

Table. Components of multidrug transport systems in selected bacterial pathogens

| Species | Pump | Efflux components: Membrane fusion protein | Efflux components: Outer membrane protein | Regulatory gene or mutation |
|---|---|---|---|---|
| Gram-negative bacteria | | | | |
| *Pseudomonas aeruginosa* | MexB | MexA | OprM | *mexR* |
| | MexD | MexC | OprJ | *nfxB* |
| | MexF | MexE | OprN | *mexT* |
| | MexY | MexX | OprM | *mexZ* |
| *Escherichia coli* | AcrB | AcrA | TolC | *acrR*, *marA*, *robA*, *soxS* |
| Gram-positive bacteria | | | | |
| *Staphylococcus aureus* | NorA | -- | -- | *flqB* promoter mutation, *arlRS* |
| *Streptococcus pneumoniae* | PmrA | -- | -- | ? |

fluoroquinolones are preferred substrates (20). It appears likely that most bacteria will have multiple MDR efflux systems.

The structural features of a fluoroquinolone that determine whether it is affected by an efflux system are not fully defined but correlate with hydrophilicity in the NorA pump of *S. aureus* (21). The risk for acquisition of resistance may be reduced for quinolones that are poor substrates for efflux pumps, since overexpression of such pumps would be unlikely to be effective as a resistance mechanism. Inhibition of pump function by other compounds is also under investigation as a means of reducing the frequency of resistance selections (22) and enhancing intrinsic activity of fluoroquinolones and other drugs that are also pump substrates.

### Other Mechanisms of Resistance

The dominant mechanisms of fluoroquinolone resistance identified are 1) chromosomal mutations causing reduced affinity of DNA gyrase and topoisomerase IV for fluoroquinolones and 2) overexpression of endogenous MDR pumps. One report, however, has documented plasmid-mediated fluoroquinolone resistance in clinical isolates of *Klebsiella pneumoniae*, transferable to *E. coli* in the laboratory (23). Neither the mechanism of this transferable resistance nor the prevalence of fluoroquinolone-resistance plasmids in clinical settings is known.

### Clinical Occurrence of Fluoroquinolone Resistance

Fluoroquinolone resistance emerged shortly after these drugs were introduced; two species were particularly affected, *S. aureus* and *P. aeruginosa*. Ciprofloxacin and ofloxacin were the most extensively used fluoroquinolones during this early period. The emergence of resistance was predicted on molecular grounds because, in these species, single mutations, which raise the MIC of ciprofloxacin of these organisms 4- to 16-fold, produce a level of resistance at or above peak drug concentrations achievable in serum, providing an opportunity for spontaneous first-step mutants to survive and emerge when a patient is exposed to fluoroquinolones. In the case of *S. aureus* and coagulase-negative staphylococci, methicillin-resistant strains developed fluoroquinolone resistance more rapidly than methicillin-susceptible strains (1,24). This difference is in part explained by nosocomial transmission in some settings and by the potential for coselection with several antimicrobial agents (because of the common multidrug resistance phenotype of methicillin-resistant strains [25]). Case-control studies have identified fluoroquinolone use as a risk factor for resistance.

Fluoroquinolone resistance has also increased substantially in some settings in species in which multiple mutational events are required for resistance to occur (e.g., *Campylobacter jejuni* [26], *E. coli* [27], and *Neisseria gonorrhoeae* [28]). Emergence in these species would not have been predicted on molecular grounds, suggesting that other epidemiologic factors may have come into play. For *C. jejuni*, resistance emerged in parallel in animal and human populations (29) shortly after fluoroquinolones were introduced for use in humans and other quinolones were introduced in food animal production, particularly poultry, in parts of Europe. In the United States, where use of quinolones in food animals was introduced later, demonstrating a link between resistant *C. jejuni* strains from poultry and food products and those causing human disease was possible (26). Thus, for a known zoonotic pathogen such as *C. jejuni*, resistance was augmented by selection pressures in an animal reservoir of campylobacters.

Fluoroquinolone resistance in *E. coli* has emerged in Europe, particularly in patients with urinary tract infections (30) and neutropenic cancer patients with bacteremia that developed during fluoroquinolone prophylaxis (31). Fecal carriage of resistant *E. coli*, however, appears to be common in both healthy adults and children in Spain (27). Carriage of resistant strains by children, a group in which fluoroquinolones are rarely used, and by adults without prior quinolone exposures (30) suggests acquisition of resistant strains by the population at large. This occurrence (in the context of documented high rates of fluoroquinolone resistance in *E. coli* isolated from poultry in Spain [32] and, by analogy, to what has been documented with campylobacters) suggests that acquisition of resistant strains from food sources may have resulted in substantial colonization of the human population with resistant *E. coli*, creating a reservoir of resistant organisms. Fluoroquinolone use in humans, which has also been shown to be a risk factor for having a resistant strain, may operate in this context to select either already fully resistant or intermediately resistant strains, accounting for the high levels of resistance and multiple mutations reported in resistant strains causing infections in humans. Whether similar problems with fluoroquinolone-resistant *E. coli* will emerge in the United States is not known, but the situation is being monitored.

Humans are the sole reservoir for infections with *N. gonorrhoeae*. In the United States, fluoroquinolone resistance in this organism has resulted largely from clonal outbreaks caused by human-to-human spread (33). Thus, for all three organisms in which fluoroquinolone resistance has become problematic despite a requirement for multiple mutations, other epidemiologic factors (of transmission and ongoing selection in reservoir populations of organisms) appear to be at work.

Newer fluoroquinolones are now incorporated into guidelines for treatment of patients with lower respiratory tract infections because of rising resistance to beta-lactams and other agents in *S. pneumoniae*, the most commonly identified bacterial pathogen in patients with community-acquired pneumonia (34). Only recently has fluoroquinolone resistance begun to emerge in this organism, albeit at low levels (2). In some cases, fluoroquinolone-resistant strains, like those resistant to beta-lactams, have emerged because of clonal spread (35). Because the newest fluoroquinolones are for treating patients with respiratory tract infections, increasing selection pressure for resistance is possible. This concern is especially great for drugs developed for use in children, who are a major reservoir of *S. pneumoniae* (36). Monitoring will be necessary, as will studies to indicate whether the improved therapeutic index for some fluoroquinolones can be translated into a lower risk of selection of resistant strains, either spontaneous or clonal, in the clinical setting.

Dr. Hooper is chief of the Infection Control Unit of Massachusetts General Hospital and program director for the Massachusetts General Hospital/Brigham and Women's Hospital Joint Fellowship in Infectious Diseases at Harvard Medical School. He is an associate professor of medicine at Harvard Medical School. His research interests include the mechanisms and epidemiology of antibiotic resistance with a particular interest in fluoroquinolone resistance.

### References

1. Hooper DC. New uses for new and old quinolones and the challenge of resistance. Clin Infect Dis 2000;30:243-54.
2. Chen DK, McGeer A, de Azavedo JC, Low DE, The Canadian Bacterial Surveillance Network. Decreased susceptibility of *Streptococcus pneumoniae* to fluoroquinolones in Canada. N Engl J Med 1999;341:233-9.
3. Low DE, Scheld WM. Strategies for stemming the tide of antimicrobial resistance. JAMA 1998;279:394-5.
4. Hiasa H, Yousef DO, Marians KJ. DNA strand cleavage is required for replication fork arrest by a frozen topoisomerase-quinolone-DNA ternary complex. J Biol Chem 1996;271:26424-9.
5. Willmott CJ, Critchlow SE, Eperon IC, Maxwell A. The complex of DNA gyrase and quinolone drugs with DNA forms a barrier to transcription by RNA polymerase. J Mol Biol 1994;242:351-63.
6. Shea ME, Hiasa H. Interactions between DNA helicases and frozen topoisomerase IV-quinolone-DNA ternary complexes. J Biol Chem 1999;274:22747-54.
7. Wetzstein HG, Schmeer N, Karl W. Degradation of the fluoroquinolone enrofloxacin by the brown rot fungus *Gloeophyllum striatum*: Identification of metabolites. Appl Environ Microbiol 1997;63:4272-81.
8. Cabral JH, Jackson AP, Smith CV, Shikotra N, Maxwell A, Liddington RC. Crystal structure of the breakage-reunion domain of DNA gyrase. Nature 1997;388:903-6.
9. Willmott CJ, Maxwell A. A single point mutation in the DNA gyrase A protein greatly reduces binding of fluoroquinolones to the gyrase-DNA complex. Antimicrob Agents Chemother 1993;37:126-7.
10. Berger JM, Gamblin SJ, Harrison SC, Wang JC. Structure and mechanism of DNA topoisomerase II. Nature 1996;379:225-32.
11. Berger JM. Type II DNA topoisomerases. Curr Opin Struct Biol 1998;8:26-32.
12. Ng EY, Trucksis M, Hooper DC. Quinolone resistance mutations in topoisomerase IV: relationship of the *flqA* locus and genetic evidence that topoisomerase IV is the primary target and DNA gyrase the secondary target of fluoroquinolones in *Staphylococcus aureus*. Antimicrob Agents Chemother 1996;40:1881-8.
13. Blanche F, Cameron B, Bernard FX, Maton L, Manse B, Ferrero L, et al. Differential behaviors of *Staphylococcus aureus* and *Escherichia coli* type II DNA topoisomerases. Antimicrob Agents Chemother 1996;40:2714-20.
14. Pan XS, Fisher LM. *Streptococcus pneumoniae* DNA gyrase and topoisomerase IV: overexpression, purification, and differential inhibition by fluoroquinolones. Antimicrob Agents Chemother 1999;43:1129-36.
15. Alovero FL, Pan XS, Morris JE, Manzo RH, Fisher LM. Engineering the specificity of antibacterial fluoroquinolones: benzenesulfonamide modifications at C-7 of ciprofloxacin change its primary target in *Streptococcus pneumoniae* from topoisomerase IV to gyrase. Antimicrob Agents Chemother 2000;44:320-5.
16. Coronado VG, Edwards JR, Culver DH, Gaynes RP. Ciprofloxacin resistance among nosocomial *Pseudomonas aeruginosa* and *Staphylococcus aureus* in the United States. Infect Control Hosp Epidemiol 1995;16:71-5.
17. Hooper DC. Mechanisms of quinolone resistance. Drug Resistance Updates 1999;2:38-55.
18. Nikaido H, Thanassi DG. Penetration of lipophilic agents with multiple protonation sites into bacterial cells: tetracyclines and fluoroquinolones as examples. Antimicrob Agents Chemother 1993;37:1393-9.
19. Bolhuis H, Van Veen HW, Poolman B, Driessen AJ, Konings WN. Mechanisms of multidrug transporters. FEMS Microbiol Rev 1997;21:55-84.
20. Köhler T, Michea-Hamzehpour M, Plesiat P, Kahr AL, Pechère JC. Differential selection of multidrug efflux systems by quinolones in *Pseudomonas aeruginosa*. Antimicrob Agents Chemother 1997;41:2540-3.
21. Yoshida H, Bogaki M, Nakamura S, Ubukata K, Konno M. Nucleotide sequence and characterization of the *Staphylococcus aureus norA* gene, which confers resistance to quinolones. J Bacteriol 1990;172:6942-9.
22. Markham PN, Neyfakh AA. Inhibition of the multidrug transporter NorA prevents emergence of norfloxacin resistance in *Staphylococcus aureus*. Antimicrob Agents Chemother 1996;40:2673-4.
23. Martínez-Martínez L, Pascual A, Jacoby GA. Quinolone resistance from a transferable plasmid. Lancet 1998;351:797-9.
24. Blumberg HM, Rimland D, Carroll DJ, Terry P, Wachsmuth IK. Rapid development of ciprofloxacin resistance in methicillin-susceptible and -resistant *Staphylococcus aureus*. J Infect Dis 1991;163:1279-85.
25. Pegues DA, Colby C, Hibberd PL, Cohen LG, Ausubel FM, Calderwood SB, et al. The epidemiology of resistance to ofloxacin and oxacillin among clinical coagulase-negative staphylococcal isolates: Analysis of risk factors and strain types. Clin Infect Dis 1998;26:72-9.
26. Smith KE, Besser JM, Hedberg CW, Leano FT, Bender JB, Wicklund JH, et al. Quinolone-resistant *Campylobacter jejuni* infections in Minnesota, 1992-1998. N Engl J Med 1999;340:1525-32.
27. Garau J, Xercavins M, Rodríguez-Carballeira M, Gómez-Vera JR, Coll I, Vidal D, et al. Emergence and dissemination of quinolone-resistant *Escherichia coli* in the community. Antimicrob Agents Chemother 1999;43:2736-41.
28. Fox KK, Knapp JS, Holmes KK, Hook EW III, Judson FN, Thompson SE, et al. Antimicrobial resistance in *Neisseria gonorrhoeae* in the United States, 1988-1994: The emergence of decreased susceptibility to the fluoroquinolones. J Infect Dis 1997;175:1396-403.
29. Endtz HP, Ruijs GJ, van Klingeren B, Jansen WH, van der Reyden T, Mouton RP. Quinolone resistance in campylobacter isolated from man and poultry following the introduction of fluoroquinolones in veterinary medicine. J Antimicrob Chemother 1991;27:199-208.

30. Ena J, Amador C, Martinez C, Ortiz de la Tabla V. Risk factors for acquisition of urinary tract infections caused by ciprofloxacin resistant *Escherichia coli*. J Urol 1995;153:117-20.
31. Pena C, Albareda JM, Pallares R, Pujol M, Tubau F, Ariza J. Relationship between quinolone use and emergence of ciprofloxacin-resistant *Escherichia coli* in bloodstream infections. Antimicrob Agents Chemother 1995;39:520-4.
32. Blanco JE, Blanco M, Mora A, Blanco J. Prevalence of bacterial resistance to quinolones and other antimicrobials among avian *Escherichia coli* strains isolated from septicemic and healthy chickens in Spain. J Clin Microbiol 1997;35:2184-5.
33. Gordon SM, Carlyn CJ, Doyle LJ, Knapp CC, Longworth DL, Hall GS, et al. The emergence of *Neisseria gonorrhoeae* with decreased susceptibility to ciprofloxacin in Cleveland, Ohio: epidemiology and risk factors. Ann Intern Med 1996;125:465-70.
34. Bartlett JG, Breiman RF, Mandell LA, File TM Jr. Community-acquired pneumonia in adults: guidelines for management. Clin Infect Dis 1998;26:811-38.
35. Ho PL, Que TL, Tsang DN, Ng TK, Chow KH, Seto WH. Emergence of fluoroquinolone resistance among multiply resistant strains of *Streptococcus pneumoniae* in Hong Kong. Antimicrob Agents Chemother 1999;43:1310-3.
36. Hendley JO, Sande MA, Stewart PM, Gwaltney JM Jr. Spread of *Streptococcus pneumoniae* in families. I. Carriage rates and distribution of types. J Infect Dis 1975;132:55-61.
